# Bilateral Post-Traumatic Facial Palsy: A Case Report and Literature Review

**DOI:** 10.22038/IJORL.2022.61355.3108

**Published:** 2022-09

**Authors:** Daniela Parrino, Roberta Colangeli, Silvia Montino, Elisabetta Zanoletti

**Affiliations:** 1 *Department of Neuroscience DNS, Otolaryngology Section, Padova University Hospital, Padova, Italy.*

**Keywords:** Facial nerve, Facial paralysis, Temporal bone fracture, Maxillofacial trauma, Skull base surgery

## Abstract

**Introduction:**

Bilateral facial nerve (FN) palsy due to temporal bone fracture is a rare clinical entity, with few cases reported. The choice between conservative and surgical treatment is more complex than in unilateral cases.

**Materials and Methods:**

A thorough search of the available literature on trauma-related bilateral FN palsy revealed 22 reports. Our own experience is also described.

**Results:**

All bilateral delayed- and unknown-onset cases were treated conservatively, with a good recovery rate (70.5%). Surgery was performed on 6 sides within the immediate-onset group, with a good recovery rate (83.3%).

**Conclusions:**

In the management of traumatic FN palsy, the main controversial issue focusses on indications for surgery as well as timing and type of approach. In bilateral cases, it is more challenging to make the right choice, due to lack of facial asymmetry and/or state of unconsciousness following severe trauma. Electro-diagnostic tests and high-resolution computed tomography are essential for decision-making.

## Introduction

Facial nerve palsy (FNP) is a relatively common neurological disorder with an incidence of 20-25 cases per million population (1). FNP typically presents unilaterally, but may also occur bilaterally, albeit rarely in around 0.3 – 2.0% of all cases, with an incidence of 1 in 5 million population (2,3). Bilateral FNP is defined as simultaneous or synchronous (when the time elapsing between paralysis on one side of the face and the onset of symptoms on the opposite side is no more than 4 weeks) or asynchronous (when the contralateral side becomes involved at a later date) (2,4). Lyme disease, Moebius syndrome, neurofibromatosis type 2, brain tumors, Melkersson-Rosenthal syndrome, and trauma are the conditions commonly underlying bilateral FNP (2).

Few cases of bilateral post-traumatic FNP have been reported in the literature, and its management remains controversial. Similarly to unilateral cases, the choice is between conservative treatment (observation and medical treatment) and surgery (which may involve surgical exploration or surgical decompression of the intratemporal extracranial and/or intracranial facial nerve) (5,6). Opting for conservative or active (surgical) treatment is not easy. Some clinical and instrumental conditions at the time of diagnosis may point in favor of observation or surgery, although the indications for one or the other are not clear-cut. It is important to balance the pros and cons for each side of the face. When observation is chosen, the clinical course of the follow-up does not prevent a shift to active treatment at a later date.

The questions to be considered are: (i) which clinical and instrumental findings at diagnosis favor immediate surgery; (ii) which clinical and instrumental parameters can drive the choice of surgery as opposed to medical treatment; (iii) what is the best surgical approach; and (iv) how to judge the benefit of active treatment over simple clinical monitoring? The literature does not provide guidelines, and clinicians have to rely on their own judgment and the approach of their local institutions. We reviewed the literature on post-traumatic bilateral FNP and report our personal experience, discussing the controversial aspects of treatment.

## Materials and Methods

*Literature review and personal experience*

A thorough search in PubMed and Scopus electronic databases was performed to identify available literature on post-traumatic bilateral FNP. First, the search terms “bilateral facial palsy”, “traumatic facial palsy” and “temporal bone fracture” were applied, and articles published in a language other than English were excluded. Any additional relevant reports mentioned in the references of the selected articles were also retrieved. In all, 32 articles were identified, but 10 were excluded due to a lack of sufficient data for the purposes of our analysis. The remaining 22 reports were considered, as summarized in table 1 (7-28). The number of cases described in each paper ranged from 1 to 3. 

Our own experience concerns a single case of a 49-year-old healthy Nigerian male presenting with post-traumatic bilateral facial palsy. The patient was admitted to our department and treated successfully, with a 12-month follow-up. He gave informed consent for clinical case publication.

## Results

*Review of the literature*

Table 1 summarizes the characteristics of the cases reported in the selected studies. The articles analyzed describe 25 patients (21 males, 4 females) with traumatic bilateral FNP, with a mean age ± SD of 29.16 ± 11.64 years (range 3 - 55 years). Road accidents were the main cause of trauma (17 patients; 68.0%), followed by blunt head traumas and high-altitude falls (4 cases each; 16.0%).

In most cases (19 patients; 80.0%), radiological findings were consistent with bilateral temporal bone fracture. Bilateral FNP was associated with a bilateral mandibular fracture in one patient. The remainder (4 patients) showed no radiological signs of fracture. Considering the 50 facial nerves separately (Table 2), the onset of paralysis was immediate in 19 cases (38.0%), and delayed in 29 (58.0%), with mean±SD time to onset of 8.24±5.71 days (range 2 - 22 days). For most patients, the paralysis reportedly occurred bilaterally at the same time. Timing of onset of bilateral paralysis was not reported in one patient (4.0%). All cases of palsy of delayed/unknown onset were treated conservatively. Surgery was performed on 6 patients in the immediate-onset group, bilaterally on two patients, and unilaterally on the other two. 

**Table 1:** Characteristics reported in the selected studies

**Author(s) (year)**	**N° cases**	**Onset**	**HB Grading**	**CT**	**MR**	**Electro-diagnostic tests**	**CSF Test**	**Treatment**	**Recovery**
Storey and Love^7^ (1949)	1	Yes	No	No	No	No	No	Yes	Yes
Cohen^8^ (1975)	1	Yes	No	No	No	No	No	Yes	Yes
Holla et al^9^ (1980)	3	Yes	No	Yes	No	Yes	Yes	Yes	Yes
Hartley^10^ (1993)	1	Yes	Yes	Yes	No	Yes	Yes	Yes	Yes
Chitkara et al.^11^ (2002)	1	Yes	No	Yes	Yes	Yes	Yes	Yes	Yes
Lee and Halcrow^12^ (2002)	1	Yes	Yes	Yes	No	No	No	Yes	Yes
Li et al.^13^ (2004)	1	Yes	No	Yes	No	Yes	No	Yes	Yes
Ulug and Ulubil^14^ (2005)	1	Yes	No	Yes	No	Yes	No	Yes	Yes
Kumar and Gupta^15^ (2006)	1	Yes	No	Yes	No	Yes	No	Yes	Yes
Roth et al.^16^ (2007)	1	Yes	Yes	Yes	No	Yes	Yes	Yes	Yes
Saidha et al.^17^ (2010)	1	Yes	Yes	Yes	No	No	No	No	No
Undabeitia et al.^18^ (2013)	1	No	Yes	Yes	No	Yes	No	Yes	Yes
Eliçora et al.^19^ (2015)	1	Yes	Yes	Yes	No	Yes	No	Yes	Yes
Kumar and Mittal^20^ (2015)	1	Yes	Yes	Yes	No	Yes	No	Yes	Yes
Kumar et al.^21^ (2015)	1	Yes	Yes	Yes	No	Yes	No	Yes	Yes
Habib et al.^22^ (2015)	1	Yes	Yes	Yes	No	Yes	No	Yes	Yes
Swain et al.^23^ (2015)	1	Yes	No	Yes	No	No	No	Yes	Yes
Salunke et al.^24^ (2016)	1	Yes	Yes	Yes	Yes	Yes	No	Yes	Yes
Ghiasi and Banaei^25^ (2016)	1	Yes	Yes	Yes	No	Yes	No	Yes	Yes
Şahin and Ӧzen^26^ (2018)	1	Yes	Yes	Yes	Yes	Yes	No	Yes	Yes
Gulati et al.^27^ (2018)	2	Yes	No	Yes	No	Yes	No	Yes	Yes
Medha et al.^28^ (2020)	1	Yes	Yes	Yes	No	Yes	No	Yes	Yes

HB: House-Brackmann Classification, CT: Computed Tomography, MR: Magnetic Resonance, CSF: Cerebrospinal fluid

**Table 2:** Patients’ paralysis characteristics. HB grade I-II is considered good recovery

**Patient ** **(gender, age)**	**Onset**		**Treatment**		**Good Recovery**	
	**Right side**	**Left side**	**Right side**	**Left side**	**Right side**	**Left side**
F, 26 ys	Immediate	Immediate	Conservative	Conservative	Yes	Yes
M, 52 ys	Delayed	Delayed	Conservative	Conservative	Yes	Yes
F, 3 ys	Delayed	Delayed	Conservative	Conservative	Yes	Yes
F, 15 ys	Delayed	Delayed	Conservative	Conservative	Yes	Yes
M, 40 ys	Delayed	Delayed	Conservative	Conservative	Yes	Yes
M, 34 ys	Delayed	Delayed	Conservative	Conservative	Yes	Yes
M, 20 ys	Delayed	Delayed	Conservative	Conservative	Yes	No
M, 29 ys	Delayed	Immediate	Conservative	Surgery	No	No
M, 16 ys	Delayed	Delayed	Conservative	Conservative	Yes	Yes
M, 24 ys	Immediate	Immediate	Surgery	Surgery	Yes	Yes
M, 21 ys	Delayed	Delayed	Conservative	Conservative	No	No
M, 27 ys	Delayed	Delayed	Conservative	Conservative	No	No
M, 45 ys	Immediate	Immediate	Conservative	Conservative	X	X
M, 38 ys	Unknown	Unknown	Conservative	Conservative	No	No
M, 40 ys	Immediate	Immediate	Surgery	Conservative	Yes	Yes
F, 22 ys	Delayed	Delayed	Conservative	Conservative	Yes	Yes
M, 27 ys	Immediate	Immediate	Conservative	Conservative	Yes	Yes
M, 23 ys	Delayed	Delayed	Conservative	Conservative	No	No
M, 22 ys	Immediate	Immediate	Conservative	Conservative	Yes	Yes
M, 26 ys	Immediate	Immediate	Conservative	Conservative	Yes	Yes
M, 35 ys	Delayed	Delayed	Conservative	Conservative	Yes	Yes
M, 32 ys	Immediate	Immediate	Conservative	Conservative	Yes	No
M, 55 ys	Immediate	Immediate	Surgery	Surgery	Yes	Yes
M, 32 ys	Delayed	Delayed	Conservative	Conservative	Yes	Yes
M, 25 ys	Delayed	Delayed	Conservative	Conservative	Yes	Yes

As shown in table 3, the recovery rate was good (House-Brackmann [HB] grade I-II), whatever the timing of onset of the palsy or treatment method involved.

**Table 3:** Rate of good recovery in relation to the onset and type of treatment of the paralysis

**Paralysis(n=50)**	**Immediate(n=19)**	**Delayed(n=29)**	**Unknown(n=2)**	**Good recovery n(%)**
Treatment				
Conservative (n=44)	13	29	2	31 (70.5%)
Surgery (n=6)	6 (2 TM, 1 MCF, 1 MCF + TM, 2 Not specified)	0	0	5 (83,3%)
Good recovery n(%)	15 (78.9%)	21 (72.4%)	0 (0.0%)	36 (72.0%)

TM: trans mastoid approach, MCF: middle cranial fossa approach

*Our experience*

**Fig 1:**
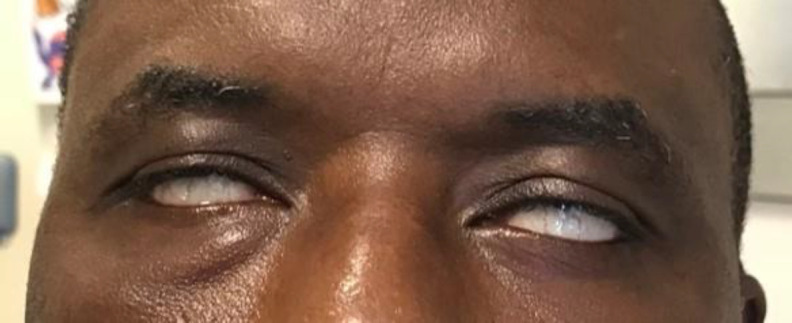
Bilateral Bell’s phenomenon

Our 49-year-old patient was admitted to the Emergency Department of Padova University Hospital after a car accident causing transient loss of consciousness in Nigeria 5 days earlier. The patient was discharged from the intensive care unit of a Nigerian hospital and transferred to Italy. He presented with diffuse upper and lower back pain, difficult and painful jaw movements, bleeding from both external auditory canals, epistaxis and bilateral, symmetrical FNP. The spine and chest X-rays identified fractures of the dorsal vertebrae and ribs, and the brain computed tomography (CT) scan showed left posterior temporal contusion and hemorrhage, and a longitudinal fracture of the right petrous bone. On otorhinolaryngological examination, otoscopy and vestibular clinical function were normal. There was no cerebrospinal fluid (CSF) otorrhea or rhinorrhea. When facial nerve function was examined, the lower portion of the face showed bilateral, symmetrical difficulty with smiling and facial expressions. In the upper portion, there was no detectable forehead movement and the eyelids did not close completely, revealing a bilateral, symmetrical Bell’s phenomenon (Figure 1). These clinical findings were consistent with a bilateral FNP, HB grade V. Audiological tests showed that hearing and tympanic membrane mobility were normal. There was a total absence of stapedius reflex bilaterally.

**Fig 2:**
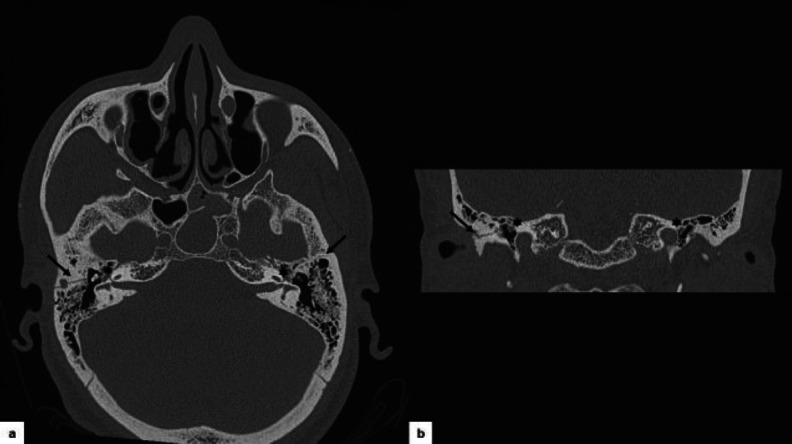
HRCT demonstrating a bilateral longitudinal fracture of the petrous bone (black arrow) with direction to the geniculate ganglion (asterisk). a. Axial section. b. Coronal section

High-resolution CT of the temporal bone identified a skull base fracture centered at the sphenoid level with a bilateral longitudinal petrous bone fracture running in the direction of the geniculate ganglion, but not clearly dissecting it (Figure 2). 

Contrast-enhanced magnetic resonance imaging (MRI) of the cerebellopontine angle showed a normal morphology and course of the VII and VIII cranial nerves. Electroneurography (ENoG) and electromyography (EMG), both obtained on day 22, showed signs of moderate-to-severe denervation bilaterally. Given the unknown time of onset of the FNP and the same HB grade on both sides, with no evidence of any electrophysiological differences, conservative management was the first choice.

After 2 months, a slight improvement in the left facial function was detectable on clinical evaluation, but high-grade paralysis persisted bilaterally. A second EMG showed denervation potentials with signs of very modest and moderate suffering on the right and left sides, respectively, and no evidence of regeneration potentials. 

Conservative management was continued for the left side, and surgery was scheduled for the right side. Eighty days after the trauma, surgical exploration was performed to expose the right geniculate ganglion region and rule out a fracture line disrupting the ganglion (as suspected on imaging). 

A middle cranial fossa (MCF) approach was chosen (29). Intraoperatively, a spicule of bone was apparent over the geniculate ganglion, along with a double fracture line from the ganglion that spread laterally towards and around the arcuate eminence. The geniculate and labyrinthine segments of the nerve were consequently decompressed. The postoperative course was free of complications and the patient was discharged 6 days after surgery. 

A month after surgery, the patient’s FNP on the right side had improved to HB grade III, at 3-month follow-up it was grade II, at 12-month follow-up it was grade I. On the left side, the patient recovered to HB grade II within 4 months after the accident, and at 12-month follow-up it was grade I. Postoperative EMG confirmed clinical improvement.

## Discussion

Post-traumatic bilateral facial nerve palsy (FNP) is a rare condition, and few cases have been reported in the literature to date. Besides those listed in table 2, other authors have described cases of bilateral traumatic FNP among their series. A first case was described by Worster-Drought in 1924 (30). In 1944, Turner identified one case of bilateral palsy among 70 cases of traumatic FNP due to closed head injuries (31). Darrouzet et al. (32) found 12 cases of facial diplegia among 115 facial nerve injuries relating to temporal bone fracture. Gaudin et al. and Wormald et al. identified another 3 and 6 cases (2,33), respectively, among their series of synchronous bilateral FNP.

Head trauma is responsible for about 5% of all cases of facial paralysis, and 3% of these are associated with temporal bone fractures (13). Temporal bone fractures are classified as longitudinal or transverse depending on the direction of the fracture line with reference to the long axis of the temporal bone. Longitudinal fractures run parallel to the long axis of the temporal bone and account for about 90% of all temporal bone fractures. In transverse fractures (the remaining 10%), the fracture line lies vertical to the petrous bone. Mixed longitudinal and transverse fractures can also occur (34). FNP accompanies approximately 30-50% of transverse fractures and 10-20% of longitudinal ones. In the former case, FNP is very serious and the prognosis often very poor, due to the high possibility of nerve transection (32). Bilateral involvement of the petrous-temporal bone is an infrequent finding, and the possible mechanism behind the production of the fracture lines was well described in 1971 by de Villiers (35). He suggested that longitudinal fractures of the petrous part of the temporal bone can cause backward displacements of the petrous apex, with coronal splitting of the body of the sphenoid, and subsequent mirror fracture lines in the opposite temporal bone, possibly leading to bilateral FNP. A transverse fracture of the petrous bone, on the other hand, will not involve the facial nerve bilaterally (35). Extraordinary cases of bilateral traumatic FNP despite apparently normal temporal bone findings on radiology have also been described (7-9,23). 

The main cause of post-traumatic bilateral FNP is reportedly severe trauma (in 68.0% of patients according to our literature review), as in the case of road accidents. Due to the high mortality and morbidity risk associated with such accidents, the condition may go unnoticed if a patient is likely to die, and its diagnosis can be particularly difficult in unconscious patients as it is impossible to appreciate any facial asymmetry. 

Traumatic FNP can be classified as immediate- or delayed-onset, depending on the time elapsing since the injury. Immediate-onset palsy is usually related to direct nerve laceration or contusion at the site of the fracture, resulting in nerve entrapment, crushing or traction. Delayed-onset palsy usually sets in 4-5 days after the trauma, when conditions such as edema, arterial or venous spasm, and hematoma may be implicated. The prognosis is generally better in the latter type of patient (14).

There are no clear guidelines available for the optimal management of this condition. Immediate-onset facial nerve injury with a fracture line running through the Fallopian canal is generally considered an indication for early surgical intervention (32). Cases of delayed-onset or incomplete facial weakness should be managed conservatively at first. Surgery may be advocated for patients failing to recover within 6 months after the trauma, but the management of such patients remains controversial (32). Since our patient could not be examined immediately after the trauma, and did not remember when his symptoms developed, we defined his palsy as being of unknown onset, and approached it as a case of delayed onset. 

For patients who are diagnosed late or whose paralysis is of unknown onset, electro-diagnostic studies (EMG and ENoG), and imaging studies are essential tools for the decision-making process. Uncontrasted high-resolution CT, with scans ideally no more than 1.5 mm apart, is the gold standard, which can clearly depict the fracture line and its relationship with the Fallopian canal. MRI with gadolinium is the method of choice for intracranial assessment: inflammatory enhancement of the distal intrameatal and labyrinthine segments can be detected, and it can be useful in preoperative examinations to rule out temporal lobe contusion, especially for MCF approaches (34).

Electro-diagnostic studies are useful for prognostic purposes as they clarify the extent of nerve damage (neuroapraxia, axonotmesis, neurotmesis). Facial nerve decompression is generally advocated if ENoG shows a >90% nerve fiber degeneration within 14-21 days after paralysis, or EMG shows no regeneration potentials after 21 days (32,36). In the case of bilateral FNP, electro-diagnostics can establish whether one side is more severely impaired than the other. The diagnostic workup adopted for this condition is still variable, however. In particular, electro-diagnostic tests are not always performed, as we can see from table 1. The same applies to MRI and CSF investigations, which are not always done, probably because they are not mandatory for surgical decision-making.

Although there are no clear-cut indications in the management of traumatic FNP, the abovementioned diagnostic issues should be considered when dealing with both unilateral and bilateral cases. In bilateral ones, each side must be considered separately to define the entity of the damage. Criteria for opting for conservative or surgical treatment should be evaluated for each side as in unilateral cases. However, the bilateral involvement leads to a lack of facial asymmetry which makes the choice of whether to treat the patient surgically or not more difficult, and, if necessary, which side to operate on first. Imaging and electrophysiological studies should be assessed carefully. In our patient, the radiologically-detected fracture line involved the geniculate bilaterally, but ENoG gave the same result bilaterally, which was not clearly suggestive of neurotmesis. Therefore, we first opted for a conservative management and follow-up. The persistence of high-grade palsy, and evidence on the second EMG of bilateral fibrillation potentials and very low response after stimulation (a pattern typical of partial high-grade nerve degeneration), worse on the right side, prompted us to undertake surgical exploration.

The choice of surgical approach is important when treating patients with facial paralysis. It depends on the site of injury and the patient’s hearing status. We chose the MCF approach to: (i) thoroughly explore the labyrinthine segment as well as the geniculate region; (ii) make a surgical graft, if necessary; and (iii) avoid manipulating the ossicular chain (as required using the transmastoid or transcanal approaches) because the patient’s hearing was intact (37). 

Timing of surgery is also important, and there is no consensus on this issue in the literature. Fisch (5) recommended urgent exploration even for delayed-onset paralysis due to possible compression by an intraneural hematoma. Other authors (14,38,39) subsequently suggested that intervention within 3 months can still be effective, and reported good outcomes in 6 - 81% of cases (HB grade I-II) even after late decompression surgery. With such findings in mind, we reassessed our patient after 2 months, and decompressed the nerve just within 3 months from trauma, obtaining a good improvement in nerve function.

In cases of severe head trauma, there may be other, concomitant cranial nerve injuries. Of course, the close relationship between the vestibular-acoustic nerve and the petrous bone, and facial nerve, mean that the former can frequently be involved as well. Temporal bone fractures are classified in otic capsule-sparing (OCS) and otic capsule-violating, depending on whether or not the capsule is affected, with injury to the vestibule-acoustic structure (40). In the series of 25 patients with bilateral traumatic facial palsy analyzed here, however, the VIII cranial nerve was only affected in 5 cases (10.0%). This may be because most of the fractures were longitudinal, which are generally OCS. In our case too, the fracture was bilaterally OCS, so the patient had no hearing impairment. The abducens nerve was often involved in the series considered here (6 cases; 12.0%), because it passes over the tip of the petrous apex. 

## Conclusion

Bilateral traumatic FNP is a rare clinical entity. Its management is controversial, its evolution is unpredictable and variable. It is not easy for clinicians to decide whether a patient should be treated conservatively or undergo surgical exploration and, if so, when, on which side and how. The diagnostic workup can be challenging due to a lack of facial asymmetry and/or a state of unconsciousness following severe trauma. Electro-diagnostic tests and high-resolution CT of the temporal bone are essential tools that aid in surgical decision-making. In the event of surgery, the MCF approach is preferable in order to: (i) explore both the geniculate and labyrinthine segment; (ii) interpose a surgical graft, if necessary; and (iii) avoid manipulating the ossicular chain in patients with intact hearing.
